# Inaction on Lead Despite the Relevant Knowledge: Predictors, Covariates, and Outreach Implications

**DOI:** 10.3390/ijerph17249391

**Published:** 2020-12-15

**Authors:** Alessandra Rossi, Bernabas Wolde, Pankaj Lal, Melissa Harclerode

**Affiliations:** 1 Department of Earth and Environmental Studies, Montclair State University—1 Normal Avenue, Montclair, NJ 07043, USA; woldeb@montclair.edu (B.W.); lalp@montclair.edu (P.L.); 2 Environmental Sustainability Scientist, CDM Smith—110 Fieldcrest Avenue, PO 8, Floor 6, Edison, NJ 08837, USA; harclerrod@cdm.com

**Keywords:** lead exposure, specialized outreach, profiled audience, risk management

## Abstract

Testing residential soil and paint for lead provides actionable information. By showing where and how much lead exists on the residence, it allows one to quantify risk and determine the best ways to reduce exposure along with the corresponding health and financial costs. For these reasons, several federal and state programs offer outreach to audiences on the benefits of testing residential soil and paint for lead. Not all individuals who know about lead’s adverse health effects, however, test their residence for lead, potentially limiting the actionable information that could have helped to reduce their exposure. Such individuals represent a challenge to outreach programs and the broader public health objectives. There is, thus, a need to understand who such individuals are and why they behave this way, allowing us to develop a specialized outreach program that addresses the problem by targeting the relevant sub-population. Using survey data, we quantitatively determine the profiles of individuals who, despite knowing about lead’s adverse health effects, are unlikely to test their residence for lead, finding statistically significant socio-economic predictors and behavioral covariates. We also find a geographic component to it, further helping outreach professionals learn how to allocate their limited resources.

## 1. Introduction

Lead is a cumulative toxicant without a safe blood concentration level [1,2,3]. Children exposed to lead can be asymptomatic at screening and early symptoms of lead poisoning can be vague [4,5]. By the time the detrimental effects of lead poisoning manifest, it may be too late as the effects are cumulative and irreversible. For these reasons, primary prevention is the main strategy for addressing lead exposure [6,7].

Proactively testing residential soil and paint for lead provides actionable information, helping one assess where and how much lead exists on the residence, determine the best ways to prevent exposure, and reduce the attendant health and financial costs [8,9]. For these reasons, several federal and state laws ban the use of lead in specific products (e.g., gasoline and paint) and provide outreach [1,4,10,11]. For example, the Residential Lead-Based Paint Hazard Reduction Act of 1992 uses information disclosure as a policy tool. By disclosing information on the sources, exposure pathways, adverse health impacts, and individuals’ rights pertaining to lead poisoning, public outreach programs aim to raise awareness, reduce information asymmetry, and correct misperceptions [12,13,14,15]. Public outreach efforts also aim to stimulate change in individuals’ attitudes and behaviors in ways that increase preparedness, which is especially valuable to the susceptible individuals or their caretakers and to the underserved sub-population who would not have that information and service otherwise [16,17,18,19].

Not all individuals who know about the adverse health effects of lead exposure and stand to benefit from the relevant legal provisions act on that knowledge, sometimes even after lead impacted material has been identified on their residence. Whereas failing to test the residence for lead due to lack of the relevant knowledge is plausible, to do so after knowing about its adverse health effects and standing to benefit from relevant legal provisions is unexpected. Such individuals’ inaction concerning lead is a health concern because the activities they engage in are not reflective of the risk they face, potentially increasing their chances of exposure.

There is, thus, a need to identify individuals who, despite knowing about lead’s adverse health effects, fail to test their residential soil and paint for lead. However, the predictors of inaction on lead and the magnitude of the problem are not fully known. Subsequently, outreach professionals do not know how to identify, meaningfully engage, and provide specialized support to these individuals. In addition, the behavioral covariates of individuals’ inaction on lead are also not fully understood. This would be useful in understanding the extent of individuals’ inaction on lead and determine the specific pathways that are at work. Such information can help outreach professionals determine the pathways they should focus on during their future outreach work and in assisting such individuals develop a management plan. These challenges affect the achievement of public health objectives, manifesting in health and financial costs that are higher than they could have been otherwise. Given the limited labor and financial resources outreach programs have and the adverse health effects of continued exposure due to individuals’ inaction on lead, such gaps in knowledge can be costly. Limited empirical analyses have, however, addressed this vulnerable sub-population, instead testing the effectiveness of outreach efforts in general and on evaluating the different modes of communicating relevant information [14,20,21].

Using Jersey City as a case study and applying a quantitative approach, we characterize such individuals to answer the following questions: How prevalent is inaction on lead? Is the limited tendency to test a property for lead specific to lead in the soil, the paint, or both? What are the attitudinal and socio-economic predictors and the behavioral covariates of the tendency to test a property for lead? What does it mean for future outreach programs?

We identify the main socio-economic and behavioral characteristics, which coupled with a geographic component help in delineating the profile of individuals who do not test their living space, despite being aware that lead affects adversely their health.

We hypothesized that various socio-economic and behavioral factors affect whether or not individuals act on their knowledge of lead’s adverse health effects. We test this hypothesis using a regression analyses and by evaluating each factor’s statistical significance. Specifically, the null hypothesis (H0) was that the socio-economic and behavioral factors considered would be statistically significant. The alternative hypothesis (HA) was that none of those factors would be statistically significant.

## 2. Background

Deterioration of lead based paint, including chipping, flaking, and peeling can make lead containing dust accessible for ingestion or inhalation, both indoors and outdoors [22,23]. Other sources of lead include water, when plumbing materials that contain lead corrode, soil contaminated from paint chips, lead deposition from car exhausts, and consumption of food grown on lead-impacted soil [1,4]. Since lead binds and accumulates in the soil, it can be persistent in the environment, impacted soil acting as a source over long periods of time [24,25]. A quarter of the U.S. residences built before 1980 have soil lead concentrations in excess of 400 parts per million (ppm), the standard for bare soil in areas where children play [1]. Blood lead level could also increase from 1 to 5 microgram per deci-liter of blood (µg/dL) for every gram of lead per kilogram (g/kg) of soil [7]. Since lead impacted particulates and soil can be tracked into residential structures from outside, individuals residing in structures not impacted by lead based paint may also be exposed to lead [26,27]. Levin et al. [7] reported that 30% of the elevated blood lead level cases in the US are from individuals who do not reside in houses with lead-based paint.

Young children under the age of three can have four to five times higher lead absorption rates than adults [28]. Due to their higher likelihood to engage in lead exposure enhancing activities such as ingesting paint chips and because of their sensitivity to its adverse effects, lead poses a serious health concern for young children. The adverse health impacts include IQ deficiency, attention and behavioral problems, and poor academic achievement [4,29,30] and subsequent long-term impacts, such as reduction in chronic neurological damage leading to potential unemployment and crime [31,32,33]. Studies have also shown that lead, along with other heavy metals found in metallurgical industries, can lead to such adverse health effects as cardiovascular diseases in middle-aged men [34]. Other studies have also show links between elevated lead concentrations in the blood and placenta of pregnant women and the occurrence of miscarriage [35].

Whereas the Residential Lead Based Paint Hazard Reduction Act requires sellers and landlords of pre-1978 houses to disclose known lead based hazards and results of reports from tests conducted before the transaction, it does not require such properties to be tested. Towards supplementing such federal laws, addressing unique challenges, and targeting the remaining gap in childhood lead poisoning, several states and municipalities have enacted or amended local lead laws regarding the inspection of such properties for lead. These local measures vary from one to another in several respects that include: age of targeted housing units, restrictions on resident characteristics, and incentives used to compel sellers and landlords to inspect their property for lead [17,18,36]. These laws, however, commonly include a version of tenant-compliant-provisions that allow residents to receive notice about lead laws, to request a free inspection from the local authority, and/or to file a non-compliance that would prompt inspection of the property and compel the landlord to address the lead hazard [36]. Owner occupants may also be able to take advantage of voluntary, free inspections [21]. In addition to these legal provisions, individuals can also get their residence inspected for a fee. Buyers, renters, and owner-occupants, thus, have opportunities to have their residence inspected for lead and to develop a management plan based on the results.

A system of outreach efforts, including municipal, state, and federal programs also work to reduce lead exposure by raising public awareness about the hazards of lead exposure, the benefits of testing residential soil and paint for lead, and individual’s rights under the laws [14,17,18,20]. Departments of Health and Human Services (DHHS), through Childhood Lead Poisoning Prevention Programs, also provide and coordinate statewide educational initiatives for high-risk communities and counties having a high number of children with elevated blood lead level (EBLL). These programs also target residential property owners and tenants, community-based organizations, child care providers, public and private building maintenance staff, and health and housing related boards and commissions using different venues including dedicated sites, at individuals’ homes, and public or private venues. Other mediums of information sharing include newsletters, leaflets, brochures, dedicated events, and targeted mailing.

The relevant laws and outreaches are, however, incomplete without a proactive engagement by buyers, renters, and owner-occupants. If individuals fail to take advantage of the legal provisions or the outreach resources available to them, they would bear health and financial costs they could have avoided. If the legislators and outreach professionals are unable to get individuals to act on lead, they are being ineffective in eliciting the desired actions, which is a challenge that needs to be investigated and addressed. Understanding why individuals fail to act on lead, despite the knowledge of its adverse health effects, and to profile such individuals for specialized outreaches are important steps towards addressing these challenges.

## 3. Materials and Methods

### 3.1. Study Area

Given its extensive and long industrial history, several non-point sources, and a large proportion of houses built before the ban on lead, New Jersey has a widespread lead pollution problem, evident in the 424 new environmental cases opened in 2014 [37]. While the percentage of children 6 to 29 months old testing blood lead concentrations at or higher than 10 µg/dL has decreased over time, remaining the same from 2013 to 2014 and slightly increasing from 2010 to 2011, the rate of decline has been slow [37].

Jersey City, the state’s second and Hudson County’s most populous city, is among the municipalities with the highest burden of lead poisoned children in New Jersey [37]. This outcome is partly explained by the relatively higher proportion of old houses and poverty in the city [38,39,40]. Compared to the state’s 30% average, 43.6% of housing units in the county, the highest in the state, were built before 1950. More than 70% of the city’s housing units were built before 1978, when the federal ban on the use of residential lead-based paint was enacted. The percentage of children younger than 5 years of age living below the poverty-line in the county is also higher than the average value for the state [37]. Consistent with these risk factors, Jersey City had the second highest percentage of new elevated blood lead level cases in 2014 as compared to other local boards of health in the state [37]. While the percentage of children younger than six testing for lead at or higher than 5 µg/dL for New Jersey is 3.15%, the value for Jersey City is 4.03%. The City also had the lowest abatement completion rate among the top five polluted counties despite having the second highest case referrals needing investigation [37].

Although the state law requires all children between 6 to 29 months to be screened, 25% of the children younger than 3 have not had their first lead screening in 2014 [37]. Despite the relatively high lead poisoning burden and being a site of active outreach, the percentage of children younger than three screened for lead in the county in 2014 was less than the state’s average. This shows why it is important that outreach programs not only raise awareness but actually produce the desired actions against lead [41].

### 3.2. Methodological Approach

We used a survey to elicit behavioral, attitudinal, and socio-economic attributes of Jersey City residents. The survey, approved by the Institutional Review Board, was administered after pilot tests and expert reviews that aimed to increase its understandability and completeness. The in-person survey, preferable to other methods in increasing response rate and survey completion, was conducted in Lincoln Park and Arlington Park. To avoid bias, we approached every third visitor we encountered in the park to participate in the study. Everyone who volunteered to do so was asked to sign a consent form and was 18 or older. Given the diversity in the area, we administered the survey both in English and Spanish, each survey lasting 23 min on average. In total, 244 usable responses were collected. We administered the survey both during weekdays and weekends at peak hours over a period of three weeks so as to maximize our reach. This also helped us ensure that we reached people who would be at work during weekdays.

The behavioral attributes elicited whether respondents engaged in activities that could expose them to lead. These activities include growing plants for consumption and decorative purposes, growing plants directly on the soil instead of using a raised bed or by purchasing topsoil for the gardening area, and whether they or their pets lay/play in the gardens/grass on the residence. The socio-economic attributes elicited by the survey included gender, ethnicity, age, type and size of household, the presence of children younger than 3 in the household, levels of income and education, employment and residency statuses, length of stay at their residence, and neighborhood of residence. The attitudinal section of the survey elicited how much risk lead represents to the respondents on a scale of one to five, ranging from “not at all risky” to “extremely risky”.

The survey also elicited if the respondents are aware of lead’s adverse health effects by using a Yes/No type of question. Moreover, the survey elicited whether or not the respondents have tested the residential soil and the paint for lead. This allows us to determine the overlap between knowing about and taking actions against lead exposure. It also allows us to identify the behavioral covariates and the socio-economic and attitudinal profiles of individuals who, despite knowing about lead’s adverse health effects, have failed to test the residential soil and paint for lead.

### 3.3. Regression Analysis

Including multiple and diverse factors in the statistical analyses of inaction on lead provides attributes that can be used to profile vulnerable sub-populations for specialized outreach. Such information can also be used to target the said individuals and allocate outreach resources efficiently. Multivariate regression can better isolate the effects of confounding variables, controlling for such relevant covariates to provide more robust results than does correlation analyses, making the sign, statistical significance, and magnitude of estimated coefficients more reliable [41]. In addition to understanding and accounting for disparities in lead exposure risk based on the variables included in the analyses, starting with a long list of potentially predictive variables also reduces the ‘missing variable’ problem and the likelihood for over/under estimating the effect of the variables included in the analyses. Given that some of the factors considered for these analyses are risk factors for elevated blood lead level, their inclusions in this analyses also helps us determine whether and how strongly these factors also predict inaction on lead.

Instead of imposing the number and structure of the explanatory variables to capture the variation in the data, we used a stepwise regression approach to iteratively determine the final models. This approach selects a subset of behavioral, attitudinal, and socio-economic variables and their respective structure in determining the regression equation best fitting the data. For a given maximum and a minimum entry and exit *p* value thresholds, this approach iteratively determines the optimal model [41].

Given the binary nature of the variable representing inaction on lead, the binary logistic regression is used to model the data. This model fits a nominal dependent response to a linear model in terms of relevant explanatory variables, Xs.

P(Y = 1│X_1_,X_2_,X_3_,X_4_,…X_n_) (1)

Log(P(Y = k_1_)/P(Y = k_2_)) = X_b_ (2)

where k_1_ and k_2_ are the two responses. The logistic function becomes:

P(Y = k_1_) = 1/(1 + e−^(α + β1X1 + β2X2 + β3X3 + …+ βnXn)^) (3)

Given the two levels, the function can be summarized as:

P(Y = k_1_) = (1 + e^−Xb^)^−1^ (4)

The regression fits a maximum likelihood procedure where the βs maximize the joint probability attributed by the model to the two options, minimizing the negative log likelihood [42].

Given (2), the odds ratio can be determined as:

Odds ratio = (Prob(Y = k_1_))/(Prob(Y = k_2_)) = exp(Xβ) = exp(β_0_).exp(β_1_ X_1_)…exp(β_i_ X_i_) (5)

## 4. Results and Discussion

### 4.1. Description of Survey Participants

This section presents results of the comparison between our survey data and Jersey City’s census data in terms of key demographic attributes, aiming to describe the respondents and to show that the data reasonably represents the city’s population. Whereas 83.68% of the survey respondents are minority, including Asian or Pacific Islander, Hispanic, and African American, 77.1% of Jersey City’s residents are ethnic minority. While 57.08% of the survey respondents are female and 48.09% of the survey respondents had a college level education, 50.6% of Jersey City residents are female and 42% of Jersey City residents had a college level education. Similarly, while 17.72% of the survey respondents have lived at their current residence for less than a year, 14.8% of Jersey City residents have lived in their current residence for less than a year. Whereas 30% of Jersey City residents are owner-occupants of their residence, 22.2% of the survey respondents are owner-occupants of their residence.

### 4.2. Subsubsection Knowing about and Testing the Residence for Lead

The results showed that individuals who test their residence for lead are statistically more likely to find that it exists in the soil and the paint, as compared to individuals who, despite knowing about lead’s adverse health effects, have not had the tests performed. Specifically, individuals who participated in testing are 18.12 and 5.37 times more likely to detect lead in the soil and the paint, respectively, as compared to individuals who, despite knowing about lead’s health effects, have not had the tests performed. Knowing the presence of lead on the residence is an important part of developing a management plan, which would allow such individuals to reduce their exposure risk. Since individuals who have not had those tests performed are not aware of the presence and severity of lead on their residence, they are ill prepared to develop a management plan and, hence, to reduce their exposure risk.

The results also showed that individuals who find lead on their residence are 5.78 and 9.58 times more likely to take exposure mitigating actions on the soil and the paint, respectively, implementing a management plan and potentially reducing their likelihood of continued exposure. In addition, individuals who test their residence for lead are up to 18.12 times more likely to find that it exists on their residence and they are, in turn, up to 9.58 times more likely to take exposure mitigating measures against lead. One wouldn’t find lead if one doesn’t test for it. However, testing for it is no guarantee that one will find it or that it will be present in alarming amounts. These results are intended to show the prevalence of lead. If considerable shares of the people who test for it do find it, then it shows a considerable prevalence that is more than mere chance. These findings suggest the possibility that a considerable share of the individuals who know about lead’s adverse health effects would find and take exposure mitigating measures against lead if they tested the residential soil and paint. Table 1 summarizes all the above results.

The results, however, did not show a statistically significant association between the knowledge of lead’s adverse health effects and a tendency to test, find, and mitigate lead exposure. Contrary to expectation, this phenomenon is not limited to a small section of the population. While nine out of ten respondents report knowing about lead’s adverse health effects, more than two thirds of them have neither tested nor taken exposure mitigating measures against lead. This result reflects both the success of outreach programs in raising public awareness and the challenge they face in turning that knowledge into action. Specifically, while 90.83% of the respondents say they are aware of the adverse health effects of lead exposure, 88.7% have not tested the soil, 67.37% not having tested the paint, and 77.02% have not taken exposure mitigating measures against lead. This shows a considerable overlap between the group of individuals who know about lead’s adverse health effects and those failing to take exposure mitigating actions against lead. The odds ratio values determining the association between knowing about lead’s adverse health effects and the likelihood of taking exposure mitigating measures against lead is statistically insignificant, the confidence intervals ranging between 0.66 and 39.42. This leads us to reject the hypothesis that inaction on lead necessarily resulted from the lack of relevant knowledge and that knowledge about lead’s adverse health effects necessarily leads one into taking actions against lead. Taken together, these results suggest the need for, at least, a two tiered outreach program, with the traditional one based on the risk factors for elevated blood lead level and the second based on individuals’ inaction on lead despite the type of knowledge traditional outreach programs create. Table 2 presents a detailed summary of the above results.

Considering that individuals who test their residence for lead are more likely to find and hence perform mitigation measures, it is reasonable to target individuals who, despite knowing about its adverse health effects, fail to test their residence for lead with a specialized outreach program. Factors can be identified that predict whether individuals, despite knowing about lead’s adverse health effects, will fail to test the residential soil and paint for lead. As presented in Section 4.3, these factors can be used to define vulnerable sub-population that should be targeted for such specialized outreach. Accordingly, two regression models are estimated, the first determining the statistical predictors of individuals who, despite knowing about leads adverse health effects, will fail to test the residential soil and the second determining the same for residential paint.

### 4.3. The Predictors and Behavioral Covariates of Inaction on Lead

#### 4.3.1. Socio-Economic Predictors of Inaction on Lead

The results of the regression analyses show statistical significance for the behavioral covariates. The results also show that several socio-economic factors significantly predict whether individuals, despite knowing about lead’s adverse health effects, will fail to test the residential paint and soil for lead (Table 3 and Table 4, respectively). The statistically significant behavioral covariates included growing plants directly on the ground without raised bed, laying/playing on the grass/garden in the residence, allowing pets lay/play on the grass/garden in the residence, and purchasing top soil for the gardening area. The statistically significant socio-economic factors included age, type of household, employment status, and neighborhood of residence.

A mosaic of socio-economic factors predicts whether individuals, despite knowing about lead’s adverse health effects, will fail to test the residential soil and paint for lead. Accordingly, individuals who are 51 and older are 3.89 times more likely to fail to test the paint on their residence as compared to individuals who are 50 and younger. Individuals who are from a two or more non-family type of household are 4.45 times more likely to fail to test the paint at their residence for lead as compared to individuals who are from household types that are either husband-wife family, male household, one-person household, or female household. Students and retirees are 4.98 times more likely to fail to test the paint on their residence for lead as compared to individuals whose employment status is either employed/self-employed, homemakers, or unemployed.

The results also show a geographic concentration of individuals who, despite knowing about lead’s adverse health effects, will fail to test the residential soil and paint for lead. Accordingly, individuals who live in the neighborhoods of Bergen-Lafayette and McGinley Square are 4.52 times more likely to fail to test the residential soil as compared to individuals who live in the neighborhoods of Hackensack Riverfront, West Side, Journal Square, and ‘Other’. Not all residents in the latter group, however, have a statistically similar tendency to test the residential soil for lead. Specifically, individuals from West Side, Journal Square and ‘Other’ neighborhoods are 11.61 times more likely to fail to test soil on their residence as compared to those who live in Hackensack Riverfront.

#### 4.3.2. Behavioral Covariates of Inaction on Lead

The results showed that some behaviors co-vary with inaction on lead. Whereas some of the significant covariates were not in keeping with the knowledge of lead’s adverse health effects, the others reflected that knowledge. Individuals who, despite knowing about lead’s adverse health effects, failed to test their residence for lead were also up to 4.29 times less likely to purchase top soil for their gardening area, up to 2.5 times more likely to lay/play in the grass/garden, and up to 3.29 times more likely to let pets lay/play in the grass/garden as compared to those who know about lead’s adverse health effects and have tested their property for lead. In addition to failing to test the residential soil and paint for lead, the behaviors such individuals engage in are not consistent with the said knowledge of lead’s adverse health effects as they may enhance the risk of exposure through direct exposure, tracking of leaded dust indoors, and exposure through the presence of lead in the produce they grow. Conversely, learning about the presence of lead, individuals who have tested the residential soil and paint are statistically more likely to purchase top soil for their gardening area, which reduces the chances of direct exposure and bioavailability of lead in the produce grown on such soil. Individuals who have tested the residential soil and paint are also statistically less likely to lay/play in the grass/garden and to let pets lay/play in the grass/garden.

Individuals who, despite knowing about lead’s adverse health effects, fail to test the paint on their residence are 2.89 times more likely to use raised gardening beds as compared to those who know about lead’s adverse health effects and have tested their residence for lead. This choice could indicate the said individual’s recognition of lead’s adverse health effects and intentions to reduce exposure. In light of the other activities they choose to engage in, however, the overall effectiveness of this single strategy in preventing lead exposure is questionable. Moreover, the additional lead exposure enhancing activities that they engage in and the others that they choose to avoid are not based on an objective assessment of where and how much lead exists on their residence, which would have informed best practices for preventing exposure. While their choice to avoid the said activities may also be fortuitous, if a concerning level of lead were to be found on their residence, it may be unnecessary. In addition, a single prevention measure may be insufficient as some of the residences may require remedial measures apart from the said behavioral choices.

Comparison of the two regression models reveals that pet interaction with the grass and gardens at a residence is the only behavioral factor that is statistically significant for exposure to lead in paint and soil. Despite this overlap, the remaining behavioral factors (i.e., planting directly in soil and purchase of top soil) are significant only in either one of the two exposure scenarios. The difference between the two sets of significant variables suggests that individuals needing specialized outreach on testing the soil and the paint are different. As such, the outreach efforts aimed at encouraging testing the soil and the paint would have to identify and target the relevant group of individuals. Matching the specialized outreach to the relevant set of audience this way may enhance the effectiveness of the programs in addressing specific needs where they exist.

Along with some of the behavioral covariates and socio-economic factors initially considered in this study, the attitudinal variables were statistically insignificant in both models. Whereas the statistically insignificant behavioral covariates included growing plants for consumption and decorative purposes, the statistically insignificant socio-economic factors included ethnicity, income, education level, gender, number of people in the household, having children younger than 3 years of age, residency status, and length of stay at the residence at the time of the survey. Although ethnicity and being a tenant significantly predicted elevated blood lead level, these factors did not significantly predict inaction on lead. Because children are sensitive and vulnerable to lead’s health effects, we had hypothesized that having children younger than 3 would significantly predict higher likelihood to test for and take exposure mitigating measures against lead. Such result is not borne by the analyses.

The statistical insignificance of these factors, however, further illustrates the need for specialized outreach. Since the predictors for elevated blood lead level and the predictors for inaction on lead do not show complete overlap, a given portion of the population relevant for the latter will not be covered under the outreach targeting the former. Considering that raising awareness itself has a limited effect in eliciting the desired lead testing practices, the specialized outreach that focuses on residential lead testing practices and targets the sub-population identified in this study may bridge this communication gap.

## 5. Conclusions

The tendency to test residential soil and paint for lead is significantly associated with finding and partaking in exposure mitigating measures against it. The knowledge about lead’s adverse health effects, however, does not significantly associate with an individual identifying or mitigating lead impacted soil and paint. Inaction on lead, thus, does not necessarily result from lack of the relevant knowledge. Individuals who do not know where and how much lead exists on their residence are also more likely to engage in activities that may increase their risk of exposure. This state of affairs highlights the need for a specialized outreach program that focuses on enabling individuals to convert their knowledge about lead’s adverse health effects into exposure preventive and mitigating actions, complementing the traditional outreach programs based on the risk factors for elevated blood lead level. Since the predictors for failing to test residential soil and paint are different from each other and from those predicting elevated blood lead level, the specialized outreach itself would have to specifically target the relevant sub-populations. In addition to the behavioral covariates and the socio-economic predictors, the geographic concentration of such individuals in given neighborhoods may also serve as a way of allocating the specialized outreach’s resources.

Despite these results, the current study isn’t without limitations that can be addressed by future studies. Such studies, for instance, can determine if the geographic concentration of individuals sharing similar lead testing tendencies applies to other study areas, pollutants, and if there is an overlap with known geographic distribution of elevated blood lead levels or incidence of other pollutants. By cross-referencing individuals’ tendency to test their residence for lead with actual blood lead concentration levels, future studies can also quantify, in blood lead level terms, the health cost of inaction on lead. Future studies can also investigate if and what types of incentives may be used in combination with information disclosure for better results. Such studies can also consider a longer list of preventive and exposure mitigating activities to determine if and why given segments of the population prefer certain type of activities as compared to others. These studies may determine the underlying reasons that explain why individuals from a given set of household types and those above a given age threshold behave in a distinct fashion compared to their counterparts, supplementing the “who” with the “why” and allowing us to better understand the mechanism involved in deciding to inspect a residential property for pollutants. Such studies can also determine the best mediums for the specialized outreach program.

## Figures and Tables

**Table 1:** Odds Ratio Results Showing Relationship between Testing for, Finding, and Acting on Lead.

		Odds Ratio	95% Confidence Interval
Soil tested for lead	Lead identified in soil	18.12	(6.08 54.00)
Paint tested for lead	Lead identified in paint	5.37	(2.08 13.85)
Lead identified in soil	Action taken against lead	5.78	(2.07 16.08)
Lead identified in paint	Action taken against lead	9.58	(3.65 25.12)

Confidence intervals that do not include one in their range are statistically significant.

**Table 2:** Odds Ratio Results Showing Relationship between Knowing about Lead’s Adverse Health Impacts and Testing for, Finding, and Acting on Lead.

		Odds Ratio	95% Confidence Interval
Know that lead can cause developmental and learning problems among children	Tested the soil for lead	2.42	(0.31 18.92)
Know that lead can cause developmental and learning problems among children	Tested the paint for lead	2.77	(0.78 9.84)
Know that lead can cause developmental and learning problems among children	Identified lead in the soil	0.63	(0.13 3.00)
Know that lead can cause developmental and learning problems among children	Identified lead in the paint	0.83	(0.17 3.88)
Know that lead can cause developmental and learning problems among children	Taken mitigating measures against lead	5.10	(0.66 39.42)

Confidence intervals that do not include one in their range are statistically significant.

**Table 3:** Inaction to Lead Paint Exposure: Summary Result for the Nominal Logistic Regression for Failing to Test for Lead in the Paint despite Knowing about the Adverse Health Effects of Lead.

Term	Estimate	ChiSquare	Prob > ChiSq	Odds Ratio
Intercept	−11.66	0.00	0.9901	-
Grow plant directly on ground without raised bed (No vs. Yes)	1.06	5.72	0.0168 ***	2.89
Family lays/plays in the garden on residence (No vs. Yes)	−0.93	4.68	0.0305 **	0.40
Have pets play in the garden (No vs. Yes)	0.76	3.22	0.0727 *	2.13
Gender (Female vs Male)	−0.33	0.91	0.3397	-
Age (50 and younger vs. 51 and older)	1.36	4.57	0.0324 **	3.89
Type of household (One person, female household, Husband-wife, Male household vs. Two or more non-family)	1.50	2.77	0.0959 *	4.45
Type of household (Male household vs. One person, female household, Husband-wife)	0.39	0.19	0.6642	-
Education (High school or less vs. Some college and above)	−0.59	2.25	0.1333	-
Education (Some college vs. college graduate)	0.56	2.02	0.1550	-
Employment status (Homemaker, unemployed, employed/self-employed vs. Student, retiree)	1.60	3.64	0.0562*	4.98
Neighborhood (Journal Square, McGinley Square vs. Hackensack Riverfront, West Side, Bergen-Lafayette, Other)	8.60	0.00	0.9927	-
Neighborhood (West Side, Bergen-Lafayette, Other vs. Hackensack Riverfront)	15.86	0.00	0.9933	-
Perceived risk of lead in paint (Not at all risky vs. Moderately weak risk)	0.45	1.41	0.2353	-

For each applicable variable, the alternate and referent groups are described in parenthesis (Alternate vs. Referent). Whole Model Test Log-Likelihood 20.10 and a *p* value of 0.0001 *. *** *p* < 0.01, ** *p* < 0.05, and * *p* < 0.1.

**Table 4:** Inaction to Lead Impacted Soil Exposure: Summary Result for the Nominal Logistic Regression for Failing to Test for Lead in the Soil despite Knowing About its Adverse Health Effects.

Term	Estimate	ChiSquare	Prob > ChiSq	Odds Ratio
Intercept	−0.94	0.58	0.4477	-
Purchase top soil for garden (No vs. Yes)	1.45	8.35	0.0039 ***	4.26
Have pets play in the garden (No vs. Yes)	1.17	5.59	0.0181 **	3.23
Ethnicity (Other vs. Caucasian, Asian or Pacific Islander, Hispanic, African American)	1.28	2.53	0.1114	-
Have children younger than 3 years (Yes vs. No)	0.86	1.54	0.2150	-
Employment status (unemployed vs. Homemaker vs. employed/self-employed, student, retiree)	0.78	1.64	0.2006	-
Neighborhood (Hackensack Riverfront, Journal Square, West Side, Other vs. McGinley Square, Bergen-Lafayette)	1.51	3.13	0.0770 *	4.52
Neighborhood (Hackensack Riverfront vs. Journal Square, West Side, Other)	2.45	3.47	0.0627 *	11.61
Neighborhood (McGinley Square vs. Bergen-Lafayette)	1.25	1.24	0.2664	-
Perceived risk of lead in soil (Not at all risky vs. Moderately risky or more)	1.01	2.40	0.1214	-

For each applicable variable, the alternate and referent groups are described in parenthesis (Alternate vs. Referent). Whole Model Test Log-Likelihood 11.723463 and *p* value of 0.0053 * *** *p* < 0.01, ** *p* < 0.05, and * *p* < 0.1.
